# Mineralization of Bone Extracellular Matrix-like Scaffolds Fabricated as Silk Sericin-Functionalized Dense Collagen–Fibrin Hybrid Hydrogels

**DOI:** 10.3390/pharmaceutics15041087

**Published:** 2023-03-28

**Authors:** Gabriele Griffanti, Marc D. McKee, Showan N. Nazhat

**Affiliations:** 1 Department of Mining and Materials Engineering, McGill University, Montréal, QC H3A 0C5, Canada; gabriele.griffanti@mail.mcgill.ca; 2 Faculty of Dental Medicine and Oral Health Sciences, McGill University, Montréal, QC H3A 0C7, Canada; marc.mckee@mcgill.ca; 3 Department of Anatomy and Cell Biology, McGill University, Montréal, QC H3A 0C7, Canada

**Keywords:** dense collagen–fibrin, hydrogels, biomineralization, osteoblastic differentiation, bone bioengineering

## Abstract

The design of hydrogels that combine both the biochemical cues needed to direct seeded cellular functions and mineralization to provide the structural and mechanical properties approaching those of mineralized native bone extracellular matrix (ECM) represents a significant challenge in bone tissue engineering. While fibrous hydrogels constituting of collagen or fibrin (and their hybrids) can be considered as scaffolds that mimic to some degree native bone ECM, their insufficient mechanical properties limit their application. In the present study, an automated gel aspiration–ejection (automated GAE) method was used to generate collagen–fibrin hybrid gel scaffolds with micro-architectures and mechanical properties approaching those of native bone ECM. Moreover, the functionalization of these hybrid scaffolds with negatively charged silk sericin accelerated their mineralization under acellular conditions in simulated body fluid and modulated the proliferation and osteoblastic differentiation of seeded MC3T3-E1 pre-osteoblastic cells. In the latter case, alkaline phosphatase activity measurements indicated that the hybrid gel scaffolds with seeded cells showed accelerated osteoblastic differentiation, which in turn led to increased matrix mineralization. In summary, the design of dense collagen–fibrin hybrid gels through an automated GAE process can provide a route to tailoring specific biochemical and mechanical properties to different types of bone ECM-like scaffolds, and can provide a model to better understand cell–matrix interactions in vitro for bioengineering purposes.

## 1. Introduction

The extracellular matrix (ECM) of bone is a highly organized, three-dimensionally (3D) patterned tissue consisting of type I collagen fibres, noncollagenous proteins, and small proteoglycans, all of which are reinforced by hydroxyapatite nanocrystallites [1]. Through provision of key structural and cell signalling cues, once secreted by osteoblasts, the collagen fibres promote cell adhesion, drive osteoblastic differentiation, and provide a substrate for biomineralization. Moreover, and predominantly attributable to its mineral phase, bone ECM is of higher stiffness compared to other connective tissues, yet it is also resorbable, thus allowing for continuous stochastic and targeted (after loading) bone remodelling [2]. Other noncollagenous biomolecules [2] contribute to various functions, such as collagen fibrillogenesis, matrix mineralization, and cell signalling, as well as the sequestration and release of growth factors and morphogens. Therefore, the design of a dynamic, bone ECM-like scaffold with an appropriate structure and biochemistry and representative biomechanical properties represents a formidable challenge. While many efforts have been directed towards the development of scaffolds that attempt to reproduce either the biochemistry and/or mechanical properties of bone ECM, limited attention has been paid to addressing both of these parameters at the same time [3].

Matrix scaffolds composed of molecules found in provisional wound healing recapitulate many of the natural processes of bone repair and regeneration [4]. In this context, the inclusion of fibrin—a provisional matrix protein that cells encounter during the wound healing process [5,6]—into scaffolds for bone tissue engineering may prove advantageous. In addition to its composition, a scaffold should be produced with a well-defined 3D geometry that facilitates mineralization, a feature which imposes certain limits on the types of materials and technologies that can be employed [7,8,9,10]. More specifically in this regard, and in order to mimic the role of noncollagenous proteins in bone ECM mineralization, a number of strategies have included negatively charged, calcium-binding bioactive molecules to promote mineralization [11].

The present study explored the use of naturally derived, dense collagen–fibrin hybrid hydrogels as potential bone-ECM-mimicking scaffolds. Reconstituted protein-based hydrogels have been widely used to mimic the native ECM structure and function in a variety of tissues [7,8,9,10,11,12]. In particular, type I collagen molecules can be extracted from tendon or skin through their dissolution in dilute acidic solutions. Through adjustment of pH and temperature to within the physiological range, procollagen molecules self-assemble into fibrous hydrogels in vitro through a process akin to in vivo fibrillogenesis [10,12]. Similarly, blood-purified thrombin and fibrinogen can undergo polymerization to generate fibrin hydrogels in vitro [13]. However, as a consequence of their highly hydrated nature, the use of both of these protein-based hydrogels as bone ECM-mimicking environments is severely limited due to a lack of structure and mechanical properties [14,15]. To this end, a number of strategies have been employed to generate densified hydrogels, thereby increasing their mechanical properties [11,16,17]. Among these, we developed the gel aspiration–ejection (GAE) method to produce both dense collagen [18] and fibrin gels [19] through the control of gel fibrillar density and alignment, with mechanical properties approaching those of native bone ECM [18,19,20].

Following our ongoing efforts to design close-to-native bone scaffolds for tissue engineering with consistent reproducibility, here we have applied an automated GAE method [19,21,22] to produce dense and aligned hybrid collagen–fibrin hydrogels with the goal of establishing appropriate mineralization patterns within the gels. To do this, the hybrid gels were functionalized with the negatively charged (rich in Asp and Glu amino acids) silk protein sericin [23]. In our previous work using sericin-loaded gels (collagen alone), incorporation of sericin accelerated acellular mineral deposition and enhanced cell-mediated mineral deposition of seeded mesenchymal stem cells [24]. In the present study, the acellular mineralization of automated GAE-generated, sericin-functionalized, dense collagen–fibrin hybrid scaffolds was assessed in simulated body fluid (SBF). Moreover, the hybrid gel effect on seeded MC3T3-E1 pre-osteoblastic cell viability, activity, and function and on cell-mediated gel mineralization was investigated in vitro for potential bone tissue engineering applications. 

## 2. Materials and Methods

### 2.1. Preparation of Highly Hydrated Gels 

Collagen gels were prepared by neutralizing solutions composed of a 4:1 volume ratio of rat tail tendon-derived type I collagen (2 mg/mL, First Link Ltd., Birmingham, UK) in 0.6% acetic acid and 10XDulbecco’s Modified Eagle Medium (DMEM, Sigma Aldrich, Oakville, ON, Canada) with 2% of 5 M NaOH (Fisher Scientific, Oakville, ON, Canada), thus resulting in a final collagen concentration of 1.6 mg/mL. Aliquots of 1.5 mL of the neutralized collagen solution were then transferred into 48-well plates and incubated at 37 °C for 30 min to induce the formation of highly hydrated collagen hydrogels.

Fibrin gels were prepared by initially dissolving fibrinogen powder (Sigma-Aldrich, Oakville, ON, Canada) in phosphate-buffered saline (PBS) to generate a stock solution (40 mg/mL) subsequently diluted in PBS to a final concentration of 1.6 mg/mL. This was then mixed with a 0.5 U/mL thrombin solution (Sigma-Aldrich, Oakville, ON, Canada), subdivided into 1.5 mL aliquots in 48-well plates, and incubated at 37 °C for 20 min to induce the polycondensation reaction of fibrinogen and thus the formation of fibrin hydrogels. Collagen–fibrin hybrid gels were prepared by mixing collagen and fibrinogen solutions at respective ratios of 2:1 and 1:1 under gentle stirring on ice. After the addition of 0.5 U/mL thrombin, the hybrid solution was subdivided into 1.5 mL aliquots in 48-well plates and gelled at 37 °C for 30 min. For sericin-functionalized gels, *Bombyx mori* (silkworm)-derived sericin powder (Sigma-Aldrich, Canada; Product number S5201) was dissolved in 10X DMEM at 40% dry weight with respect to collagen [24].

### 2.2. Preparation of Dense Gels through Automated GAE

As-cast, highly hydrated gels were processed through automated-GAE [19,21] to generate cylindrically shaped dense collagen, fibrin, and collagen–fibrin (+/− sericin) gels. A syringe pump (diluter unit, GeSim mbH, Dresden, Germany) filled with an incompressible fluid (water or PBS) necessary for gel ejection was used to apply pressure differentials. All gels were aspirated using an 8G needle attached to a syringe pump, which generated negative pressure to draw the gels into the needle. Once the gels were drawn into the needle, positive pressure was applied to eject the dense gels.

### 2.3. Cell Culture Seeding of MC3T3-E1 Pre-Osteoblasts

Cells from the commercially available passage 16 MC3T3-E1 Subclone 14 mouse (C57BL/6) pre-osteoblast cell line (ATCC^®^, CRL2594™, Manassas, VA, USA) were incubated at 5% CO_2_ and grown to 80% confluency at 37 °C. The expansion and growth medium consisted of alpha-MEM (Gibco, Grand Island, NJ, USA) containing 1% penicillin/streptomycin (Gibco, Canada), 2 mM L-glutamine (Gibco, Grand Island, NJ, USA), 1 mM sodium pyruvate, and 10% *v*/*v* of foetal bovine serum (HyClone Laboratories Inc., Logan, UT, USA). Cell seeding was carried out post-collagen solution neutralization (as described above) at a density of 3 × 10^5^ cells/mL. Subsequently, aliquots were cast into 48-well plates and incubated in a 5% CO_2_ atmosphere at 37 °C for 30 min to induce gel formation prior to automated GAE. Cell-seeded gels were cultured for up to 35 days in growth medium (basal medium), growth medium supplemented with 50 μg/mL ascorbic acid to enhance collagen synthesis, and osteogenic medium consisting of growth medium supplemented with ascorbic acid plus 10 mM β-glycerophosphate as an organic phosphate source (Sigma, Oakville, ON, Canada) to enhance mineralization. Cell culture media were replaced at two-day intervals.

### 2.4. Seeded Cell Viability and Metabolic Activity

Confocal laser scanning microscopy (CLSM; Carl Zeiss LSM510, Toronto, ON, Canada) was used to image the viability/mortality of seeded MC3T3-E1 cells in gels at days 1 and 21 of culture. Seeded cells were stained with 2 μM calcein AM and 4 μM Ethidium homodimer-1 (Life Technologies, Toronto, ON, Canada) solution (i.e., in culture medium) and incubated at 37 °C for 30 min prior to viewing. Excitation by an argon laser (488 nm) allowed for detection of green fluorescence detection in live calcium-laden cells, while excitation by a HeNe laser (543 nm) allowed for red fluorescence detection, reflecting nuclear content in compromised and dead cells. 

Cell metabolic activity was assessed using the AlamarBlue^®^ assay (Life Technologies, Toronto, ON, Canada). MC3T3-E1 pre-osteoblasts seeded into gels (n = 3) were stained in growth medium with 10% AlamarBlue^®^ reagent and incubated under darkness in 5% CO_2_ at 37 °C for 4 h. A fluorescent detection system was employed using a Mitras LB 940 microplate reader (Berthold Technologies, Baden-Wüttemberg, Germany) equipped with a 555/580 nm filter pair. The background fluorescence measured in the medium incubated with acellular gels was subtracted from all values. Measurements were acquired at days 1, 7, 14, and 21 in culture, with the data being normalized against the fluorescent intensity at day 1.

### 2.5. Alkaline Phosphatase (ALP) Activity and Mineral Ion (Ca and P) Quantification

ALP activity was used as an indicator of osteoblastic differentiation for the seeded MC3T3-E1 cells. Gels were washed three times with PBS, then transferred into a 10 mM Tris, pH 7.4, 0.2% IGEPAL and 2 mM phenylmethylsulfonyl fluoride (all Sigma-Aldrich, Oakville, ON, Canada) solution on ice. Gels were sonicated and centrifuged at 10,000× *g* for 10 min at 4 °C to obtain cell lysates. ALP activity was measured in the cell lysates using SIGMAFAST™ p-nitrophenyl phosphate tablets (Sigma-Aldrich, Oakville, ON, Canada) as per the manufacturer’s instructions. ALP from bovine intestinal mucosa (Sigma-Aldrich, Oakville, ON, Canada) was used as a standard, and ALP activity was normalized against cellular protein content and measured at days 3, 21, and 35 of culture.

Mineral deposition in cell-seeded gels was quantified at day 35 of culture by dissolving the pelleted mineral with 0.5 M HCl at 4 °C for 1 h. Calcium and phosphorus content from the dissolved mineral was measured spectrophotometrically using calcium and phosphorus assay kits (Sekisui Diagnostic, Burlington, MA, USA).

### 2.6. Acellular Mineralization in SBF

The extent of acellular mineralization in neat and sericin-functionalized dense collagen–fibrin gels was assessed by their conditioning in Kokubo’s SBF [25]. Using a standardized ratio of 10:1 (mL/mg) SBF:gel (pH 7.45 ± 0.05 at 37 °C), mineralization was investigated at days 3, 7, and 14. The solution was replaced at two-day intervals by fresh, filtered SBF.

### 2.7. Mechanical Testing

The mechanical properties of the as-made automated-GAE gels were characterized through tensile testing using a Univert tester (CellScale Biomaterials, Waterloo, ON, Canada). Tests (n = 5) were carried out using a 10 N load cell at a displacement rate of 0.1 mm/s. Stress–strain outputs were generated from initial load–displacement data by using the needle internal diameter (3.43 mm corresponding to an 8G needle) as the nominal diameter of each gel and initial specimen length, respectively. The ultimate tensile strength (UTS) and the apparent modulus were calculated by using the maximum load and slope of the linear region, respectively, of the stress–strain curves.

### 2.8. Attenuated Total Reflectance-Fourier Transform Infrared (ATR-FTIR) Spectroscopy

ATR-FTIR spectroscopy was used to characterize the as-made gels, gels post-conditioning in SBF, and cell-seeded gels post-culturing in osteogenic medium. Samples were rinsed three times with deionized water and then freeze-dried for 24 h at −56 °C and 18 mTorr (FreeZone benchtop freeze dry system, Labconco, Kansas City, MO, USA). Spectra were collected (Spectrum 400, Perkin Elmer, Hopkinton, MA, USA) at a resolution of 2 cm^−1^, an infrared range of 4000–650 cm^−1^, and 64 scans. Spectra were then corrected with a linear baseline and normalized (absorbance of Amide I at 1643 cm^−1^ = 1.5) using Spectrum software (Perkin Elmer, Hopkinton, MA, USA).

### 2.9. Scanning Electron Microscopy (SEM)

SEM analysis was used to morphologically characterize the as-made gels, gels post-conditioning in SBF, and cell-seeded gels post-culturing in osteogenic medium. Gels were fixed in 4% paraformaldehyde for 30 min, rinsed with PBS for 10 min and then dehydrated through an ethanol gradient followed by immersion in 1,1,1,3,3,3-hexamethyldisilazane (Sigma-Aldrich, Oakville, ON, Canada) and air-dried. All samples were coated with Pt using a Leica Microsystems EM ACE600 sputter coater (Wien Wien, Austria). Images were acquired with an FEI Inspect F- 50 FE-SEM (FEI Company, Hillsboro, OR, USA) operating at 5 kV. Energy-dispersive X-ray spectroscopy (EDX/EDS) was performed at 10 kV to chemically characterize the cell-mediated mineral deposition at day 35 in osteogenic medium.

### 2.10. X-ray Diffraction (XRD)

XRD was used to characterize the as-made gels, gels post-conditioning in SBF, and cell-seeded gels post-culturing in osteogenic medium. XRD diffractograms of freeze-dried gels (as described above) were recorded using a Bruker D8 Discover diffractometer varied from 6 to 60° 2θ at 40 kV and 40 mA. Three frames of 30° were recorded for 10 min and then merged during data post processing. The phase composition was determined by comparing the acquired diffraction pattern with peaks identified in the International Centre for Diffraction Data (ICDD) database.

### 2.11. Micro-Computed Tomography (Micro-CT)

Micro-CT was used to visualize the formation of a dense mineral phase within the entire volume of the acellular gels at day 14 in SBF and in cell-seeded gels at day 35 in osteogenic medium, respectively. Freeze-dried samples (as described above) were analysed with a Zeiss Xradia Versa 520 (Zeiss, Göttingen, Germany) at 60 kV and 73 µA, with no filter and a 7 µm pixel size. Sample volumes were then reconstructed (Dragonfly software, ORS Inc., Montreal, QC, Canada) and the two phase densities observed were highlighted in blue and green, respectively.

### 2.12. Statistical Analysis

Statistical analysis was performed using a multiple *t*-test with a significance level equalling 0.05.

## 3. Results

### 3.1. Gel Preparation and Characterization

**Figure 1:**
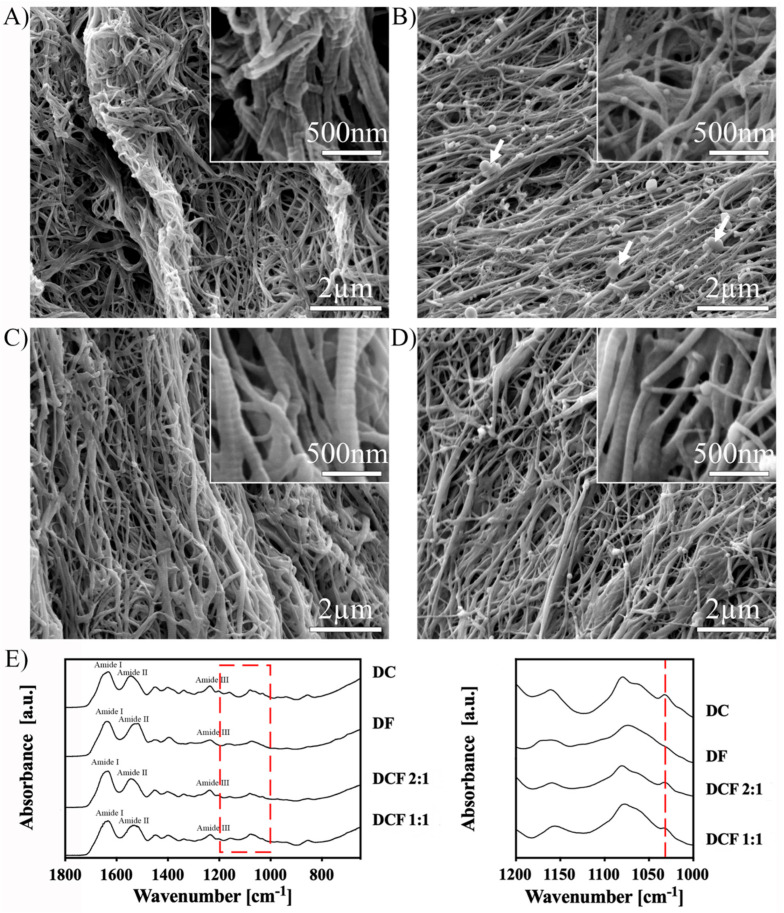
Characterization of as-made GAE-generated DC, DF, and DCF gels. SEM micrographs of (**A**) DC gels demonstrating the typical *d*-banding (inset) of type I collagen fibrils, (**B**) DF gels demonstrating a combination of smooth fibres and clots, (**C**) DCF 2:1, and (**D**) DCF 1:1 hybrid gels showing entanglements of both fibrous types. Insets showing higher magnification images. (**E**) Left panel: ATR-FTIR spectra of as-made DC, DF, and DCF gels. Spectra of DCF gels appeared similar to those of DC gels. Right panel: expansion of the area indicated by the red dashed rectangle in the left panel. The intensity of the absorption band at 1035 cm^−1^ (red dashed line) observed in the spectrum of DC gels was absent in that of DF gels and decreased with increasing fibrin content in the hybrid gels (right).

Automated GAE was applied on as-made precursor highly hydrated hydrogels to generate cylindrically shaped densified gels. SEM micrographs of the dense collagen (DC) gels revealed the typical bundling of the *d*-banded collagen fibrils (Figure 1A), whereas those of the dense fibrin (DF) gels indicated more individually organized smooth fibres (Figure 1B). These respective features were maintained through hybridization in both dense collagen–fibrin 2:1 and 1:1 (DCF 2:1 and 1:1, respectively), with intermixed fibrous entanglements (Figure 1C,D). ATR-FTIR spectroscopy allowed for the structural characterization of the gels (Figure 1E). In DC and DCF 2:1 gels, the amide I and II absorption bands at 1643 and 1525 cm^−1^, respectively, confirmed the presence of the collagen triple-helix, while the amide III absorption band was detected at 1233 cm^−1^. In the DF gels, the amide I, II, and III bands were identified at 1650, 1530, and 1230 cm^−1^, respectively (Figure 1E, left). The absorption band at 1035 cm^−1^ observed in the DC spectrum was not present in the DF gel (Figure 1E, right panel), and its intensity decreased with increasing fibrin content in the DCF gels (Figure 1E, right panel). On the other hand, the main spectral absorption bands of the DC and DF gels almost overlapped, and DCF hybrid gels did not display other substantial differences. 

**Figure 2:**
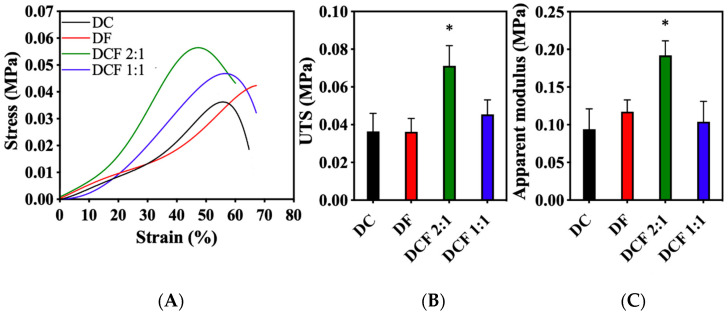
Mechanical properties of the as-made GAE-generated DC, DF, and DCF gels. (**A**) Representative stress–strain curves, (**B**) ultimate tensile strength, and (**C**) apparent modulus of DC, DF, DCF 2:1, and DCF 1:1 gels. The ultimate tensile strength and apparent modulus were significantly (* *p* < 0.05) higher in DCF 2:1 versus all other gel types.

Tensile testing allowed for mechanical characterization of the gels (Figure 2). Stress–strain curves revealed typical soft tissue-like responses to tensile testing, as defined by three distinct regions: toe, linear, and failure segments. Compared to DC, DF, and DCF 1:1 gels, DCF 2:1 gels demonstrated significantly higher (*p* < 0.05) ultimate tensile strength (UTS) and apparent modulus (AM) values. Based on these elevated UTS and AM values, the potential of the DCF 2:1 hybrid +/− sericin as bone-ECM-mimicking scaffolds was further investigated in this study.

### 3.2. Acellular Mineralization of Sericin-Functionalized DCF Gels in SBF

**Figure 3:**
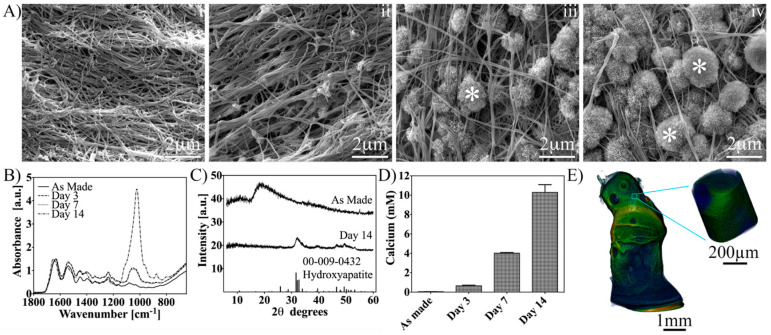
Characterization of acellular mineralization of sericin-functionalized DCF 2:1 gels up to day 14 in SBF. (**A**) SEM micrographs of as-made gels (i) and at days 3 (ii), 7 (iii), and 14 (iv) in SBF. Spherulitic mineral deposition (asterisks) was detected amongst the fibres between days 3 and 14 in SBF. (**B**) ATR-FTIR spectra of gels as a function of time in SBF. There was an increase in the absorption bands related to phosphate and carbonate groups with time in SBF. (**C**) XRD diffractograms of as-made gels and at day 14 in SBF. The appearance of a peak at 32 2 theta degrees suggested the presence of an apatitic phase at day 14 in SBF. (**D**) Calcium assay of gels up to day 14 in SBF. The amount of calcium extracted from gels increased with time in SBF. (**E**) Micro-CT 3D reconstruction of the gels at day 14 in SBF. A mineralized phase (green) can be distinguished from the organic nonmineralized phase (blue) within the gels. Inset, higher magnification of a partially mineralized volume of the gel. SEM micrographs and ATR-FTIR spectra of neat DCF 2:1 gels up to day 14 in SBF are given in Figure S2.

The acellular mineralization of the silk sericin-functionalized hybrid (DCF 2:1 SS) gels was compared to that of neat DCF gels for up to 14 days in SBF. Sericin functionalization of the gels was initially confirmed by the methylene blue binding assay (Figure S1). SEM micrographs of DCF 2:1 SS indicated a time-dependent increase in mineral deposition, which was initiated on day 3 (Figure 3A). ATR-FTIR spectroscopy revealed a temporal increase in the absorption bands attributable to phosphate (ν_1_ at 1070 and 1033 cm^−1^ and ν_3_ at 957 cm^−1^) and carbonate (ν_3_ at 1450 and 1420 cm^−1^ and ν_2_ at 872 cm^−1^) groups (Figure 3B), characteristic of carbonated hydroxyapatite. In contrast, neat hybrid gels did not display any mineral deposition up to day 14 in SBF (Figure S2A), while displaying a negligible increase in the absorption bands related to phosphate and carbonate groups (Figure S2B). XRD diffraction of the sericin-functionalized gels confirmed the presence of an apatite-like phase on day 14 (Figure 3C), where the characteristic peaks of a hydroxyapatite pattern at 32, 47, and 54 2θ degrees were detected. The progression of bulk mineralization quantified by a biochemical assay for calcium showed an increasing amount of calcium up to day 14 in SBF (Figure 3D). Correlating to this, 3D imaging by micro-CT on day 14 in SBF revealed the presence of mineralized (highlighted in green) and a nonmineralized (highlighted in blue) phases in the DCF 2:1 SS gel (Figure 3E).

### 3.3. Seeded MC3T3-E1 Cell Viability, Morphology, Proliferation, and Osteogenic Differentiation

**Figure 4:**
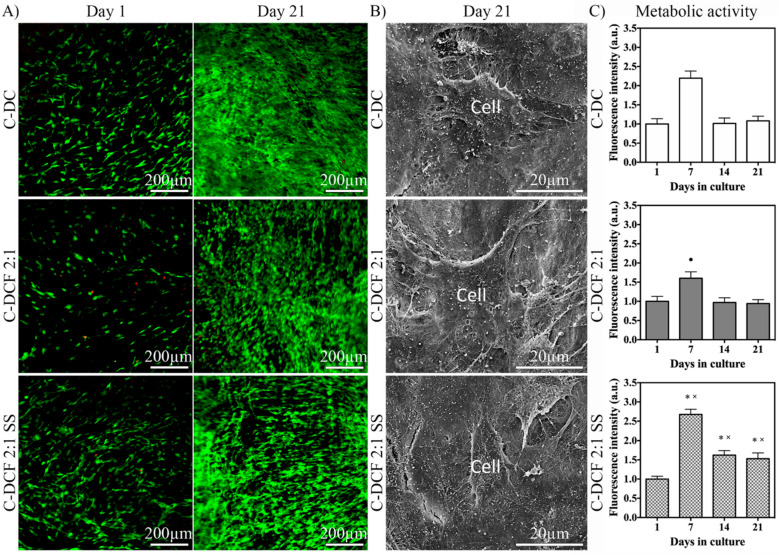
Seeded MC3T3-E1 pre-osteoblast viability, morphology, and metabolic activity under osteogenic medium. (**A**) Live/Dead™ assay of MC3T3-E1 cells cultured in DC, DCF 2:1, and DCF 2:1 SS gels at days 1 and 21. Cells grew both in time and space with negligible mortality and no qualitative difference among the three gels. (**B**) SEM micrographs of seeded MC3T3-E1 cells. Cells appeared to be well-spread and anchored to all gel types. (**C**) Metabolic activity of MC3T3-E1 cells in DC, DCF 2:1, and DCF 2:1 SS gels cultured up to day 21 in osteogenic medium. • indicates significantly (*p* < 0.05) lower metabolic activity compared to that of cells seeded in DC gels. * indicates significantly (*p* < 0.05) higher metabolic activity compared to that of cells seeded in DC gels. × indicates significantly (*p* < 0.05) higher metabolic activity compared to that of cells seeded in DCF gels.

The function of MC3T3-E1 pre-osteoblastic cells when seeded in DCF 2:1 SS gels was compared to those seeded in neat DC and DCF 2:1 gels. CLSM of calcein AM- and EthD-1-stained MC3T3-E1 cells at days 1 and 21 grown in osteogenic medium demonstrated extensive cell viability in all gel types (Figure 4A), and SEM micrographs confirmed their close association and attachment to the gels through extensive cell projections (Figure 4B). The cell metabolic activity, measured through the AlamarBlue^®^ assay, under osteogenic medium conditions, indicated a trend defined by three phases for all gels, which was characterized by an initial increase up to day 7, followed by a decrease at day 14, and finally a plateau up to day 21 (Figure 4C). The metabolic activity of cells seeded in DCF 2:1 was significantly (*p* < 0.05) lower than that of cells seeded in DC at day 7, whereas cells seeded in DCF 2:1 SS were of significantly (*p* < 0.05) higher metabolic activity than those seeded in DC and DCF 2:1 at days 7, 14, and 21 (Figure 4C).

**Figure 5:**
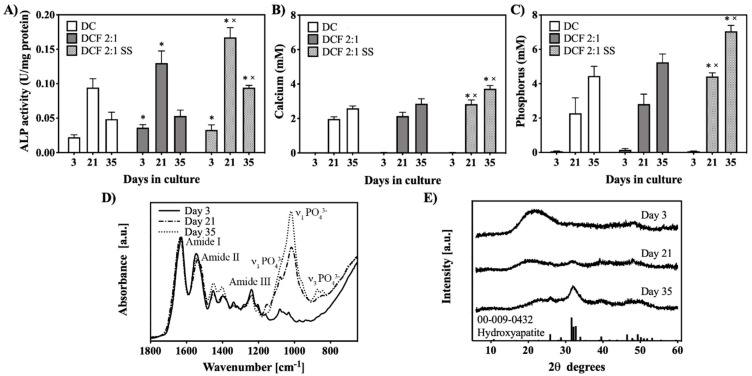
Osteogenic differentiation of MC3T3-E1 cells seeded in gels cultured up to day 35 in osteogenic medium. (**A**) ALP activity in cells seeded in DC, DCF 2:1, and DCF 2:1 SS at days 2, 21, and 35 in culture. ALP activity followed a similar trend in all gels, demonstrating an increase in activity between days 2 and 21, followed by a decrease at day 35. * indicates significantly (*p* < 0.05) higher ALP activity compared to that of DC. × indicates significantly (*p* < 0.05) higher ALP activity compared to that of DCF 2:1. (**B**,**C**) Quantification of calcium and phosphorus in medium post-mineralization. (**D**) ATR-FTIR spectra of cell-seeded DCF 2:1 SS gels as a function of culture time in osteogenic medium. There was an increase in the absorption bands related to phosphate and carbonate groups with time in culture. (**E**) XRD diffractograms of cell-seeded DCF 2:1 SS gels as a function of culture time in osteogenic medium. The appearance of a peak at 32 2 theta degrees suggested the presence of an apatitic phase at day 35 in culture.

Osteogenic differentiation was investigated through ALP activity. Cells seeded in all gels demonstrated an increase in ALP activity up to day 21, followed by a decrease at day 35 (Figure 5A). Compared to those seeded in DC, cells seeded in DCF 2:1 exhibited significantly higher (*p* < 0.05) expression of ALP activity at days 3 and 21. Cells seeded in DC 2:1 SS expressed significantly higher (*p* < 0.05) ALP activity compared with those seeded in DC at all time points and at days 21 and 35, when compared with those seeded in DCF 2:1 (Figure 5A). The decrease in ALP activity in all gels at day 35 corresponded to an increase in mineralization, as measured through calcium and phosphorus biochemical assays (Figure 5B,C). The amounts extracted from DCF 2:1 SS were significantly higher (*p* < 0.05) than those extracted from the other two gels at days 21 and 35 (Figure 5B,C). The ALP activities of cell-seeded gels cultured in basal medium were characterized by a different trend compared to those cultured in osteogenic medium, with a slight increase at day 35 and no significant differences among all gels (Figure S3A). On the other hand, calcium and phosphorus were not detected in cell-seeded gels cultured in basal medium (Figure S3B,C). ATR-FTIR spectra of cell-seeded DCF 2:1 SS indicated increased intensities in absorption bands related to phosphate and calcium groups with time in culture under osteogenic medium (Figure 5D). XRD diffractograms of DCF 2:1 SS indicated the presence of an apatite phase at the latter time point (Figure 5E).

**Figure 6:**
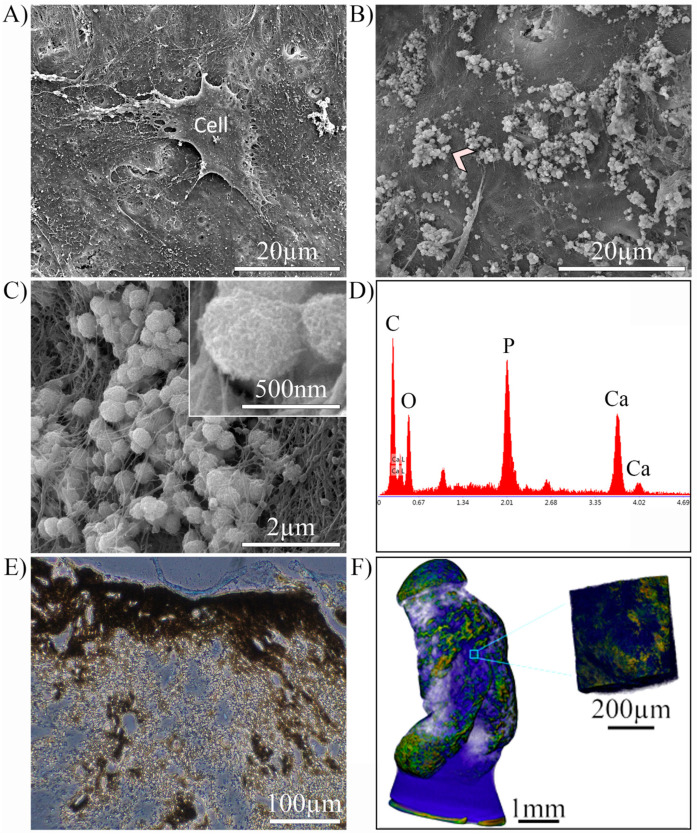
Characterization of MC3T3-E1 cell-mediated mineralization of DCF 2:1 SS gels at day 35 in osteogenic medium. (**A**) SEM micrograph of a seeded MC3T3-E1 pre-osteoblastic cell cultured in osteogenic medium. (**B**) SEM micrograph of mineralized matrix (chevron). (**C**) Higher magnification of the matrix area indicated by the chevron in (**B**). Inset, higher magnification images of the spherulitic-shaped apatite mineral amongst the fibres. (**D**) EDX analysis of the area indicated by the chevron in (**B**) detecting the presence of calcium and phosphorus. (**E**) von Kossa staining for minerals in representative histological sections of seeded gels. Blue spots are cells while brown/black areas are minerals. (**F**) Micro-CT 3D reconstruction of a mineralized gel at day 35. A mineralized phase (green) can be distinguished from organic phase (blue) within the gel. Inset, higher magnification of a partially mineralized volume of the gel.

The MC3T3-E1 cell-mediated mineral deposition in DCF 2:1 SS at day 35 under osteogenic medium was further characterized morphologically and chemically (Figure 6). SEM (Figure 6A) revealed flattened osteoblastic cells anchored to the scaffold through extensive cell projections. Higher magnification images showed cell-mediated mineral deposition through extensive apatite-like spherulitic particle deposition and growth (Figure 6B,C), which corroborated the XRD diffractograms. SEM-EDX analysis of mineral particles identified calcium and phosphorus at a ratio of 1.65 ± 0.05, this being consistent with the presence of an apatitic mineral phase (Figure 6D). von Kossa (silver nitrate) staining of gel histology sections for light microscopy identified heterogeneous regions of mineralized DCF 2:1 SS (brown/black, Figure 6E), consistently associated with regions of high osteoblast density. In 3D, reconstruction after micro-CT imaging likewise confirmed the heterogeneity of the mineralized regions within the gels (Figure 6F).

## 4. Discussion

This work demonstrates that automated-GAE-enabled dense collagen–fibrin hybrid gel scaffolds functionalized with silk sericin create a bone ECM-like microenvironment, which undergoes mineralization in SBF in the absence of cells and supports osteogenic cell differentiation from pre-osteoblasts seeded in three dimensions within the scaffolds.

Ideally, an ECM-like scaffold aims to promote the restoration of damaged tissue by controlling cellular functions and guiding their spatial and temporal activities that lead to tissue formation and regeneration [9]. Control of mineralization in such scaffolds may additionally be desirable for mineralized tissue applications, particularly for bone treatments. For these reasons, the ability to mimic the structure or function of the native ECM microenvironment through provision of appropriate molecular cues is critical in the design of a successful scaffold [26]. For mineralized tissue applications, since the ECM of bone is dominated by a hierarchically organized fibrous network [27,28], the fibrillar components of the collagen–fibrin gels were chosen based on the target cell function—those of the osteogenic lineage. Type I collagen provides innate biological cues to promote cell adhesion, proliferation, orientation, and chemostatic response [26]. Fibrin, on the other hand, facilitates cell proliferation, migration, and differentiation [29]. In the context of adding another functionalizing protein to the scaffolds, we selected silk sericin on the basis that it likewise supports cell adhesion, viability, and proliferation [24,30], while also acting as an osteostimulating biomolecule [24].

Although both collagen and fibrin gels have been used separately as scaffolds to support various cellular activities [31,32,33,34], their highly hydrated nature results in a lack of structural and mechanical properties that are needed to more closely resemble a bone ECM-like environment [9,14]. Accordingly, in previous work, we therefore explored various strategies to improve the mechanical properties of either collagen or fibrin gels [11]. For example, plastic compression has been used extensively to generate dense collagen gels of randomly oriented fibres with improved mechanical properties without compromising seeded cell viability and proliferation [16,17]. On the other hand, while it is known that the mechanical properties of fibrin gels can be increased with increasing fibrinogen concentration [35,36], highly concentrated fibrinogen solutions cannot be readily sterilized, thus limiting their cellular applications. To circumvent this, a plastic compression technique was developed that enables the design of cell-receptive compacted fibrin gels with improved mechanical properties [17]. Automated GAE is a recently developed alternative route for fabricating dense collagen and fibrin gels characterized by predictable mechanical properties [18,21]. The GAE method has been shown to be a powerful tool to reproduce well-defined 3D geometries and fibrillar orientation, which not only mimic the structural and mechanical cues of native bone ECM, but also support cell proliferation and osteoblastic differentiation in vitro [18,19,20] and mineral formation in vivo [37]. Accordingly, in the present study, we used a highly reproducible automated GAE technology design to generate collagen–fibrin hybrid scaffolds to mimic the bone ECM microenvironment and which were additionally functionalized by incorporating another bioactive molecule, silkworm sericin. In support of our approach, work by others using highly hydrated collagen–fibrin gels mixing 1.5 mg/mL collagen with either 5, 10, or 25 mg/mL of fibrin led to an increase in mechanical properties at higher fibrin concentrations [38]. In this same study, although it was also shown that an increase in mechanical properties supported the growth of seeded human mesenchymal stem cells up to day 14, the work did not report on their osteogenic differentiation [38].

The interplay between ECM stiffness and compliance has been shown to regulate cellular function and morphogenesis [39]. For example, the functionality of MC3T3-E1 pre-osteoblastic cells has been reported to be enhanced when seeded in substrates of greater stiffness [40]. Induction of cultured MC3T3-E1 pre-osteoblast cells along their osteoblast (and even osteocyte) lineage occurs through a differentiation pathway that leads to a very close approximation of native bone in terms of ECM production and mineralization [41]. In the present study, it was demonstrated that an increase in gel stiffness favourably influenced the proliferation and osteogenic differentiation of these MC3T3-E1 osteoblast-lineage cells. Moreover, along with an apparent increases in modulus, the strength of the hybrid gels also increased with increases in collagen content, suggesting complex interactions between the two fibrillar networks [42]. In the case of these hybrid gels, it is likely that close, noncovalent interactions and entanglements not only increased the total fibrillar density, but also enhanced the mechanical properties of the gels through a stiffening mechanism at higher strains [43].

The ability of our hybrid gels to undergo mineralization in the absence of cells was assessed in SBF, as performed previously [11]. As part of the present work, we found that the incorporation of sericin accelerated the mineralization process. Sericin, while occurring in a number of forms, is rich in acidic amino acids (aspartic acid and glutamic acid) [23] and serine (a potential phosphorylation site), features similar to many biomineralization-regulating proteins thought to act through their calcium-binding properties in regulating mineralization trajectories [11]. More specifically, such proteins (particularly osteopontin) [44] appear to use poly-acidic stretches of amino acids to stabilize mineral precursor phases prior to crystallization in a process called the PILP process, ultimately facilitating collagen mineralization in vitro [45,46].

Pre-osteoblastic cells, seeded in 3D throughout the gels as part of the processing route (as opposed to layering), were found to be adherent, viable, proliferative, and well distributed within the gels. After an initial increase up to day 7, the metabolic activity decreased at day 14, beyond which its level was maintained up until day 21. This decrease may be attributed to cell–cell contact inhibition [47,48], as indicated by CLSM images at day 21, which showed fully colonized gels. The metabolic activity of cells seeded in DCF 2:1 was lower than those in DC alone, possibly due to its higher density and stiffness, which are known to decrease cell proliferation [40,49,50]. On the other hand, sericin incorporation into DCF 2:1 enhanced cell proliferation, as previously demonstrated [24,30].

The osteogenic outcome of MC3T3-E1 cell seeding was assessed through measurements of ALP activity as well as by assessments of ECM mineralization. The activity of ALP, which correlates with osteoblast differentiation and the production of a mineralizable ECM by these cells, can be considered as an early marker of mineralization [51,52,53]. This activity peaked on day 21 and was followed by a decrease on day 35, which coincided with increases in both calcium and phosphorus content in the scaffolds, quantified as an indication of mineralization within the gels over time in culture. The hybrid gels were more mineralized compared to DC gels alone, suggesting that the hybrid gels could be more effective in supporting osteoblastic growth and differentiation [54]. This is in agreement with previous findings showing that denser and stiffer scaffolds with preferential fibril alignment promote the osteogenic differentiation of seeded cells [20,55]. Moreover, the incorporation of sericin led to a significant increase in calcium and phosphorus deposition, which appeared to be directionally governed from the exterior to interior of the gels, as indicated by both histological staining and micro-CT reconstruction. This may be attributable to cell growth being mostly in the 200–400 µm peripheral layer of the gel, as dictated by concentration gradients associated with the mass transport of oxygen, nutrients, and catabolites [56]. This suggests that cells located in the outer layer of the gels were first to undergo differentiation, depositing ECM that mineralized, in which they were subsequently embedded. This process decreased towards the inner region of the gels, where networks of cells were observed with irregular and more sporadic areas of mineralization. Once fully differentiated, osteoblasts acquire the ability to secrete mineralized matrices, and ultimately, they become trapped in their own mineralized matrix as osteocytes [57] to act as mechanosensors (among other functions) as a final positioning process related to this cell lineage.

In summary, the in vivo osteoblastic differentiation pathway is characterized by distinct stages composed of cell proliferation and differentiation, followed by ECM deposition and mineralization [58,59]. Notably, in our study here, the seeded MC3T3-E1 cells appeared to go through these stages when seeded in all the gel/scaffold types we used. However, seeded cell proliferation, ALP activity, and mineral deposition showed differences in the various scaffolds. In particular, while the growth of cells seeded in DCF 2:1 was reduced in comparison to those seeded in DC, the differences in osteogenic differentiation and mineral deposition were marginal. On the other hand, the sericin-functionalized DCF 2:1 scaffold ultimately resulted in accelerated osteoblastic differentiation and greater mineral deposition, and this formulation may thus meet the needs of a tissue that requires the rapid stimulation of ECM and mineralization. Thus, these findings offer promise for the design of specialized scaffold to tailor the needs of specific bone therapies and applications.

## 5. Conclusions

This study has shown that an automated GAE fibre alignment process can successfully generate dense collagen–fibrin hybrid gels with a fibrous structure more closely emulating bone ECM than many other methods currently in use. Moreover, varying their composition with reproducible flexibility in the scaffold design offers specific tailoring of the distinct structural and mechanical properties. The functionalization of these scaffolds with silk sericin enabled their acellular mineralization in simulated body fluid and promoted the proliferation and differentiation of seeded MC3T3-E1 pre-osteoblastic cells, which in turn led to a greater extent of cell-mediated matrix mineralization. Therefore, the ability to tailor the scaffold structure and properties to better imitate the native bone ECM, with the addition of cell seeding, provides a route to not only design a scaffold that meets the needs of a specific bone defect, but to also better understand the processes regulating cell–matrix interactions.

## Data Availability

Not applicable.

## Supplementary Materials

The following supporting information can be downloaded at: https://www.mdpi.com/article/10.3390/pharmaceutics15041087/s1, Figure S1: Macroscopic images of DCF gels *post*-methylene blue staining. Only the sericin-functionalized DCF gels allowed for dye binding (blue/purple staining). Figure S2: Characterization of mineral formation in neat DCF 2:1 gels. Figure S3: ALP activity, calcium, and phosphorus quantification in gels seeded with MC3T3-E1 cells and cultured under basal medium.
