# Supplementary material for: Mineralization of Bone Extracellular Matrix-like Scaffolds Fabricated as Silk Sericin-Functionalized Dense Collagen–Fibrin Hybrid Hydrogels
Source: Pharmaceutics. 2023 Mar 28;15(4):1087. doi: 10.3390/pharmaceutics15041087 (PMC10142947; doi:10.3390/pharmaceutics15041087)
Supplement: Supplementary file 1 [file pharmaceutics-15-01087-s001.zip › pharmaceutics-2301724-supplementary.pdf]

Supporting information

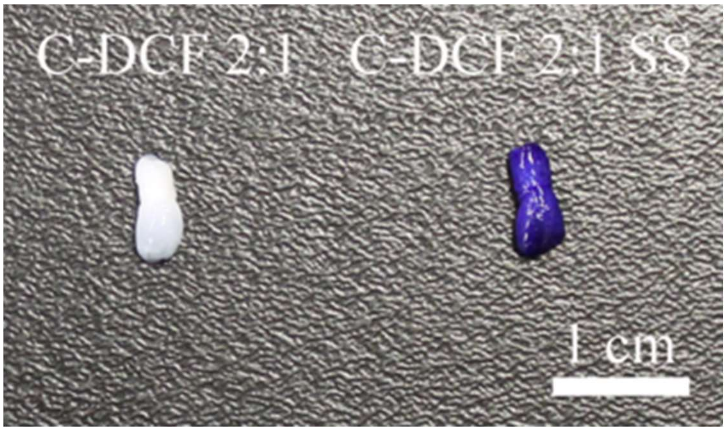

Figure S1. Macroscopic images of DCF gels *post*-methylene blue staining. Only the sericin-functionalized DCF gels allowed for dye binding (blue/purple staining).

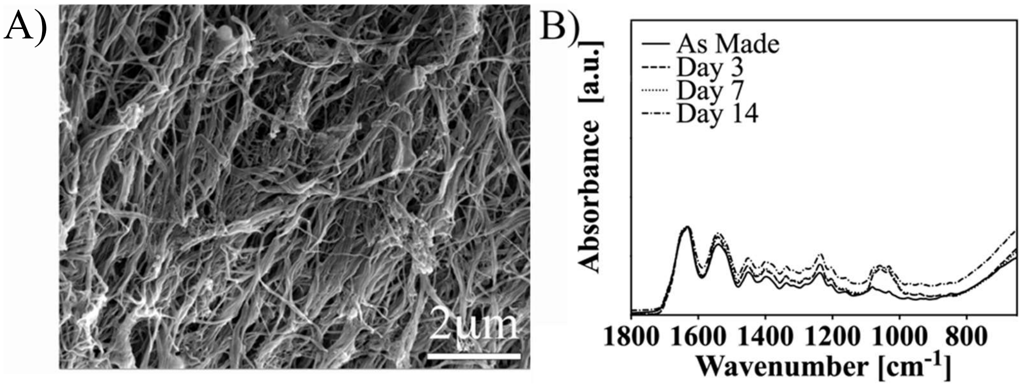

Figure S2. Characterization of mineral formation in neat DCF 2:1 gels. A) SEM micrographs at day 14 in SBF. No mineral deposition was observed. B) ATR-FTIR spectra as a function of time in SBF. There was no detectable increase in absorption bands related to phosphate or carbonate groups as a function of time in SBF.

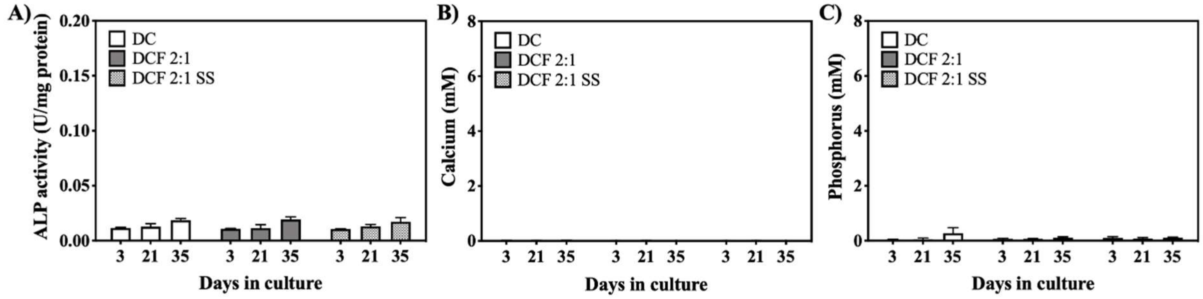

Figure S3. ALP activity, calcium, and phosphorus quantification in gels seeded with MC3T3-E1 cells and cultured under basal medium. A) ALP activity. There was no significant ($p > 0.05$) difference between the gels at the various time points. Residual B) calcium and C) phosphorus quantification in culture medium indicating no mineralization.
